# Quality of life and associated factors among family caregivers of individuals with psychiatric illness at DRH, South Wollo, Ethiopia, 2020

**DOI:** 10.1038/s41598-022-22015-4

**Published:** 2022-11-03

**Authors:** Habtam Gelaye, Atsedemariam Andualem

**Affiliations:** 1 Department of Psychiatry, College of Medicine and Health Sciences, Wollo University, 1145 Dessie, Ethiopia; 2 Department of Nursing, College of Medicine and Health Sciences, Injibara University, Injibara, Ethiopia

**Keywords:** Health care, Quality of life

## Abstract

Mental illness results in an enormous social and economic burden not only on patients, but also on their families and communities. Many caregivers of patients with mental illnesses suffer from an extremely poor quality of life. In Sub-Saharan Africa, approximately 71% caregivers suffer from economic burden of severe mental illness. To our knowledge, no study has been conducted on quality of life of caregivers in Ethiopia. Therefore, this study aimed to assess the quality of life of family caregivers of patients with mental illness at Dessie Referral Hospital. The institution-based cross-sectional study was conducted among 398 caregivers selected using a consecutive sampling technique. The World Health Organization Quality of Life BREF was used to assess the quality of life. Logistic regression was performed and statistical significance was declared at a p-value < 0.05. 189 (47.5%) of family caregivers had poor quality of life. Being divorced, unable to read and write, primary education, being spouse, sibling and children of the patient, poor social support and high perceived stigma were significantly associated with the outcome variable. Because the magnitude of poor quality of life among family caregivers was high, family intervention programs are highly recommended to improve quality of life among caregivers.

## Introduction

Mental illness is a condition characterized by significant disturbances in cognitive, emotional, and behavioral functioning. Common mental illnesses include schizophrenia, depression, bipolar disorder, and anxiety disorders^1^. Studies have shown that approximately 450 million people are affected by mental illness and their devastating effects at personal and national levels are quite significant^2,3^.

According to the World Health Organization (WHO), quality of life (QOL) is defined as an individual’s perception of their position in life in the context of the culture and value systems in which they live and in relation to their goals, exceptions, standards and concerns. This definition considers an individual’s satisfaction with the physical, psychological, social, environmental and spiritual aspects of life^4,5^.

According to systematic review conducted in Sub-Saharan Africa, five out of seven studies (71%) estimated the full economic burden of severe mental illness on caregiver^6^. Individuals suffering from mental illness, their families, and their communities face a massive social and economic burden^2^.

According to a study conducted in Iran in 2018, not meeting the needs of caregivers, burnout, high burden of care, high social stigma, low social support, and low QOL were among the main challenges faced by caregivers^7^. This is supported by studies conducted in Uganda and Indonesia, which found that 57.3%^8^ and 36.2%^9^ of caregivers had a poor quality of life, respectively.

A study conducted in Nigeria revealed that caregivers of patients with mental illness face a multidimensional range of problems, often associated with their caregiving role^10^. A study conducted in Italy found that caregivers who care for patients with mental illnesses had significantly lower QOL^11^. As evidenced by different studies, family caregivers’ QOL may have a direct impact on the worsening of symptoms of patients and indirectly on patients’ QOL. That means it is the fundamental of success in patients’ recovery and plays an important role in preventing relapse and also readmission^9,12,13^.

Previous studies focused on caregivers’ burden, which is the only form of pain experienced by caregivers of patients with mental illness. However, they did not address stigma, clinical factors, substances, and social exclusion from society. Therefore, this study focused on addressing these gaps and assessing whether caregivers were emotionally, physically, economically, or socially affected.

In Ethiopia, although numerous studies have been conducted on the QOL of patients with mental illness, the caregivers’ QOL and its associated factors remain unexplored. To fill this gap, this study sought to determine the prevalence and identify factors associated with the quality of life of caregivers of patients with mental illnesses at Dessie Referal Hospital (DRH).

## Materials and methods

### Study area and study period

The study was conducted at Dessie Referral Hospital (DRH) psychiatric outpatient clinic from January1 up to February 30, 2020. DRH is found in Dessie Town, South Wollo Zone, Amhara Region, Ethiopia. Dessie is located 400 km northeast of Addis Ababa, the capital city of of Ethiopia. It is the only referral hospital in Wollo province that provides services for a catchment area population of 8 million people, including the neighboring regions. The study was conducted in a psychiatric outpatient clinic that provides services for approximately 800 patients and has three outpatient departments with nine staff members.

### Study design

Institutional based cross sectional study was conducted.

#### Source population

- All family caregivers of patients with mental illnesses on follow up at a psychiatric clinic at DRH.

#### Study population

- Family caregivers of patients with mental illnesses who visited a psychiatric clinic during the study period.

#### Inclusion criteria

- Being identified by the individual with mental illness as a family caregiver
- Individuals who were caregivers of patients diagnosed with mental illness for at least six months
- Caregivers who were willing to participate in the study
- Caregivers who were ≥ 18 years of age

#### Exclusion criteria

- Caregivers who were diagnosed with severe mental illness or who had aggressive or the potential to harm themselves or people around them and those who were unable to stay around the clinic long enough to complete the tool.

### Sample size and sampling procedure

The sample size was calculated using the single-population proportion formula. Since there is no similar study conducted in Ethiopia and to obtain the maximum sample size, we took prevalence of poor quality of life of caregivers of patients with mental illness (p) 50% with 95% CI and 5% margin of error. The sample size was calculated as follows:

$$\begin{aligned} {\text{n}} &= \, \left( {{\text{Z }}\alpha /{2}} \right)^{{2}} {\text{p }}\left( {{1} - {\text{p}}} \right) /{\text{d}}^{{2}} \hfill \\ {\text{n }} &= \, \left( {{1}.{96}} \right)^{{2}} \,\left( {0.{5}0} \right) \, \left( {{1} - 0.{5}0} \right)/ \, \left( {0.0{5}} \right)^{{2}} \hfill \\ &= { 384}.{16}\sim { 384} \hfill \\ \end{aligned},$$

where n = minimum sample size, Z α/2 = 1.96, p = prevalence of QOL 50%, d = margin of error (5%).

We added 10% to the calculated sample size for the possibility of non-respondents; the final sample size for the study was 423.

A consecutive sampling technique was used to select participants. Caregivers of patients with mental illnesses who visited a psychiatric clinic were recruited consecutively.

### Data collection tools and techniques

Data were collected through face-to-face interview using a structured Amharic version questionnaire by four trained BSc psychiatric professionals and two supervisors over a period of two months (January 1 to February 30, 2020) after obtaining written consent from the respondents. The questionnaire consisted of six parts, with the first part being sociodemographic, the second part was about clinical factors of the patient and the others were WHOQOL-BREF, Devaluation of Consumer Family Scale (DCFS), Oslo 3-items Social Support Scale and Alcohol, Smoking and Substance Involvement Screening Test (ASSIST**)**.

*The WHOQOL-BREF* is the most widely used health-related quality of life measurement tool in the world^5^. It was developed by WHO. It contains 26-items with four domains: physical health (7 items), psychological health (6 items), social relationships (3 items), and environmental health (8 items). It also contains items that measure general perceptions of life and health (Items 1 and 2, respectively). Each item of the WHOQOL-BREF is scored on a Likert scale from 1 (very dissatisfied) to 5 (very satisfied). The score ranges from 26 (lowest score) to 130 (highest score). The mean of each domain and the mean total score were also calculated. The Mean WHOQOL-BREF score was used to categorize QOL as good or poor. Hence, subjects who scored less than or equal to the mean score were categorized as having a poor QOL and those who scored greater than the mean score were categorized as having a good QOL^14^. The internal consistency in this study was Cronbach α = 0.886.

*The Devaluation of Consumer Family Scale (DCFS)* was used to assess stigma among family caregivers of patients with mental illnesses. It has nine items rated on a four-point Likert scale (1 = strongly disagree, 4 = strongly agree) and three items (DCFS2, DCFS5 and DCFS6) are negatively worded. The three negatively worded items were reverse coded to obtain a higher DCFS score representing a higher level of perceived stigma^15^. Internal consistency (Cronbach’s alpha) was 0.85.

*The Oslo 3-items Social Support Scale* was used to assess the social support status of the family caregivers. A sum index was created by summarizing the raw scores, with the sum ranging from 3 to 14, categorized as 3–8 = poor support, 9–11 = moderate support and 12–14 = strong support^16^.

*The Alcohol, Smoking and Substance Involvement Screening Test (ASSIST)* was developed under the auspices of the World Health Organization (WHO) by an international group of addiction researchers. It is an 8- item questionnaire designed to be administered by health workers to a client and takes approximately 5–10 min. ASSIST was designed to screen different substances and the sum ranging 0–10 = low risk, 11–26 = moderate risk and ≥ 27 = high risk for alcohol and 0–3 = low risk, 4–26 = moderate risk and ≥ 27 = high risk for other substances. The score obtained for each substance falls into a ‘lower’, ‘moderate’ or ‘high’ risk category which determines the most appropriate intervention for that level of use (‘no treatment’, ‘brief intervention’ or ‘referral to specialist assessment and treatment’ respectively)^17^.

### Study variables

#### Dependent variables

- **Quality of Life (Good/Poor)**

#### Independent variables

- Sociodemographic factors, clinical factors, social support, perceived stigma and history of substance use

### Operational definitions

- **Family caregiver:** A family member or relative who has the most frequent contact with the patient; provides unpaid support to the patient financially, socially, psychologically, and physically; and has mostly been collateral in the patient’s treatment^18^.
- **Mental illness**: A condition characterized by significant disturbances in cognition, emotional regulation, and behavioral functioning, such as schizophrenia, depression, bipolar disorder, anxiety disorders, etc^19^.
- **Quality of life:** Caregivers who scored less than or equal to the mean score were categorized as having POOR QOL and those who scored greater than the mean score were categorized as having GOOD QOL^14^**.**
- **High-perceived stigma**: According to Devaluation and Consumer Family scale (DCFS) those family caregivers who scored greater than mean were considered as having high-perceived stigma.
- **Substance use:** By using Alcohol, Smoking and Substance Involvement Screening Test (ASSIST V3.0).
- For alcoholic beverages, those who scored 0–10 were low risk, 11–26 were moderate risk and ≥ 27 were high risk, indicating that they experienced severe problems (health, social, financial, legal and relationships) as a result of their current pattern of use and were likely to be dependent^17^.
- For tobacco products, cannabis, cocaine, amphetamine-type stimulants, inhalants, sedatives and pills, hallucinogens, opioids and other substances, those who scored 0–3 were at low risk, 4–26 were at moderate risk and ≥ 27 were at high risk of experiencing severe problems (health, social, financial, legaland relationship) as a result of their current pattern of use and were likely to be dependent^17^.
- **Social support:** According to the Oslo-3 Social Support Scale (OSS-3), family caregivers who scored 3–8 had poor support, 9–11 had moderate support and 12–14 had strong support^16^.

### Data quality control

Four BSc psychiatry professionals were hired for data collection and two master-level mental health professionals were hired to supervise the data collectors. Data collection tools were first prepared in English, translated into local languages (Amharic) and then re-translated back into English by another person who was blinded to the English version to check the clarity of the questionnaire. The data collectors underwent one-day training on the questionnaire and the method of assessment. Pre-test was conducted two weeks before the start of actual data collection to determine the time needed to complete one questionnaire, to determine whether the questionnaire used was understandable to the study participants and to see its consistency.

### Data processing and analysis

First, the data were coded and entered into Epi Data 3.1 then exported to the Statistical Package for Social Sciences version 20 (SPSS-20) for analysis. Descriptive statistical analysis was used to estimate the mean, frequency, and percentage of variables. Binary logistic regression analysis was performed and variables with a p-value < 0.25 were eligible for multivariable logistic regression analysis. After multivariable logistic regression analysis, the adjusted odds ratio with 95% CI was used to determine the significance association between the outcome and explanatory variables. Variables with a p-value less than 0.05 were considered statistically significant.

### Ethics approval and informed consent

Ethical approval was obtained from the Institutional Review Board of Wollo University**.** The participants were informed of the study objectives before the interview began. Written informed consent was obtained from all participants. All methods were performed in accordance with relevant guidelines and regulations.

## Results

### Socio-demographic characteristics of respondents

The total response rate in this study was 94.1%. Among a total of 398 respondents, 246 (61.8%) were male. The mean age of participanst was 37.43 years (SD ± 12.853). More than half of the study subjects 231 (58.0%) were married. The majority of participants were Amhara 365 (91.7%) in ethnicity and Muslim 298 (74.9%) in religion. Almost half of the study participants were living in urban areas 216 (54.3%). Concerning living with the ill relatives, the majority of the participants 352 (88.4%) were living with an ill person. The average monthly income of the participants was 3942.78 ETB (SD ± 14,286.176) (Table 1).

**Table 1:** Distribution of participants by socio-demographic characteristics of participants (n = 398) at DRH, Ethiopia, 2020.

Variables	Variable category	Frequency	Percent (%)
Age	18–37	217	54.5
	≥ 38	181	45.5
Gender	Male	246	61.8
	Female	152	38.2
Marital status	Married	231	58.0
	Single	105	26.4
	Divorced	35	8.8
	Widowed	27	6.8
Ethnicity	Oromo	14	3.5
	Amhara	365	91.7
	Tigray	5	1.3
	Other^a^	14	3.5
Religion	Muslim	298	74.9
	Orthodox	79	19.8
	Protestant	17	4.3
	Other^b^	4	1.0
Residency	Urban	216	54.3
	Rural	182	45.7
Educational status	Unable to write and read	81	20.4
	Primary	114	28.6
	Secondary	104	26.1
	College and above	99	24.9
Occupation	Governmental worker	79	19.8
	Farmer	92	23.1
	Merchant	30	7.5
	house wife	79	19.8
	daily laborers	31	7.8
	Other^c^	87	21.9
Monthly income	≤ 3942 ETB	291	73.1
	> 3942 ETB	107	26.9
Relationship with the patient	Parent	81	20.4
	Spouse	89	22.4
	Siblings	101	25.4
	Children	61	15.3
	Others^d^	66	16.6
Living with the ill relatives	Yes	46	11.6
	No	352	88.4

Other^a^ includes (Guragie).

Other^b^ includes (catholic religion).

Other^c^ includes (students, jobless or unemployed).

Others^d^ includes (aunt, uncle, grandfather and grandmother).

### Substance related characteristics

The majority of study participants 330 (82.9%) used one or more than one substances in their lifetime. Out of these, the majority of study participants 282 (70.9%) used khat in their lifetime. Regarding the level of risk to health, the majority of them were at low risk of cannabis 373 (93.7%), followed by tobacco products 372 (93.5%), alcohol beverages 366 (92.0%) and khat 224 (56.3%) (Table 2**).**

**Table 2:** Substance related characteristics of family caregivers of patients with mental illness (n = 398) at DRH, 2020 (n = 398).

Variables	Variable category	Frequency (n)	Percent (%)
Substance use	No	68	17.1
	Yes	330	82.9
Life time use of tobacco	No	360	90.5
	Yes	38	9.5
Life time use of alcohol beverage use	No	308	77.4
	Yes	90	22.6
Life time use of cannabis	No	373	93.7
	Yes	25	6.3
Life time use of khat use	No	116	29.1
	Yes	282	70.9
Tobacco	Low risk	372	93.5
	Moderate risk	22	5.5
	High risk	4	1.0
Alcohol beverage use	Low risk	366	92.0
	Moderate risk	18	4.5
	High risk	14	3.5
Cannabis	Low risk	373	93.7
	Moderate risk	25	6.3
Khat use	Low risk	224	56.3
	Moderate risk	92	23.1
	High risk	82	20.6

### Clinical factors about the patient and social support of the caregivers

Regarding clinical factors of the patient, 236 (59.3%) of study participants’ duration of the illness was ≤ 5 years; nearly half of them 184 (46.2%) had continuous episodes and 288 (72.4%) had ≤ 5 years duration of treatment. Majority of study participants 328 (82.4%) had no previously diagnosed comorbid medical illnesses. More than half of the participants 214 (53.8%) reported poor social support Table 3).

**Table 3:** Clinical factors about the patient and social support related factors among caregivers of patients with mental illness at DRH, Ethiopia, 2020 (n = 398).

Variables	Variable category	Frequency (n)	Percent (%)
Duration of illness	≤ 5 years	236	59.3
	> 5 years	162	40.7
Frequency of episodes	1. Continuous	184	46.2
	2. Two	152	38.2
	3. > 2	62	15.6
Duration of treatment in year	≤ 5 years	288	72.4
	> 5 years	110	27.6
Diagnosed co morbid medical illness if any?	1. Yes	70	17.6
	2. No	328	82.4
Social support	Poor support	214	53.8
	Moderate support	120	30.2
	Strong support	64	16.1

### Perceived stigma characteristics

The mean DCFS score was 22.08 with SD ± 2.48. More than half of the study participants 233 (58.5%) had low perceived stigma and about 165 (41.5%) had high perceived stigma.

### Quality of life among family caregivers of patients with psychiatric illness

In this study, the mean score of the participants’ overall QOL was 80.06, with SD ± 13.82. Nearly half of the participants (47.5%, n = 189 and (95% CI = 41.2, 51.5) scored below the mean, while the remaining 52.50% (n = 209) scored above the mean were poor and good quality of life, respectively. The WHOQOL BREF also covers four different domains of QOL (physical, psychological, social relationships, and environmental). Among the four domains of QOL, the participants scored the highest mean in the physical domain (26.57 ± 4.80) and the lowest mean in the social relationship domain (8.696 ± 2.59) than the other domains (Table 4).

**Table 4:** The mean distribution across the four domains of WHOQOL-BREF and the over all QOL of caregivers of patients with mental illness, DRH, Ethiopia.

WHOQOL-BREFF domains	Mean (± SD)	Range	
		Minimum	Maximum
Physical domain	26.57 ± 4.80	14	35
Psychological domain	17.93 ± 3.13	7	25
Social relationship domain	8.696 ± 2.59	3	15
Environment domain	21.397 ± 5.59	10	35
Over all QOL	80.06 ± 13.82	40	115

### Self-rated percieved quality of life and health satisfaction among family caregivers of patients with mental illness at DRH

The study participants were asked to provide their perception of their quality of life and health satisfaction. Based on their perceived quality of life, nearly half (48.7%) of study participants reported their perceived quality of life was neither poor nor good, followed by 124 (31.2%) who reported poor. Concerning their perceived satisfaction with their health, 182 (45.7%) study participants were neither dissatisfied nor satisfied, and nearly one third (124, 31.2%) were dissatisfied (Table 5).

**Table 5:** Self-rated perceived quality of life and health satisfaction among family caregivers of patients with mental illness at DRH, Ethiopia (n = 398).

Variable	Variable category	Frequency (n)	Percent (%)
Perceived quality of life	Very poor	26	6.5
	Poor	124	31.2
	Neither poor nor good	194	48.7
	Good	48	12.1
	Very good	6	1.5
Perceived health satisfaction	Very dissatisfied	24	6.0
	Dissatisfied	124	31.2
	Neither dissatisfied nor satisfied	182	45.7
	Satisfied	60	15.1
	Very satisfied	8	2.0

### Factors associated with quality of life among family caregivers of patients with psychiatric illness

We performed bivariate and multivariable regression analysis to identify factors associated with the caregivers’ quality of life. Variables with a p-value < 0.25 in the bivariate analysis were included in the multivariable analysis by the backward method. The final multivariable logistic regression model showed that marital status, educational status, relationship with the patient, social support, and perceived stigma were found to be statistically significant for QOL at the level of p-value < 0.05.

The odds of having poor QOL among divorced caregivers of patients with mental illness were nearly 3 times higher [AOR = 2.921, 95% CI 1.256, 6.794] as compared to married participants. Furthermore, the odds of having poor QOL were 6 times higher among participants who were unable to write and read [AOR = 6.176, 95% CI 2.872, 13.278] and 3 times higher among participants educated up to primary school level [AOR = 3.129, 95% CI 1.659, 5.900] than those educated at college and above levels. Furthermore, spouses of patients were 2.4 times more likely to have poor QOL [AOR = 2.407, 95% CI 1.088, 5.322], siblings of patients were 2.4 times more likely to have poor QOL [AOR = 2.422, 95% CI 1.143, 5.135], and children were nearly 2.5 times more likely to have poor QOL [AOR = 2.543, 95% CI 1.105, 5.853] than others. Besides, participants who had poor social support were 3.2 times at higher odds of having poor QOL [AOR = 3.206, 95% CI 1.578, 6.511] than those who had strong social support. And participants who had high perceived stigma were 2.4 times at higher odds of having poor QOL [AOR = 2.376, 95% CI 1.489, 3.792] than those who had low perceived stigma (Table 6).

**Table 6:** Adjusted multivariable analysis model showing independently associated factors with QOL among caregivers of patients with mental illness (n = 398).

Variables	Variable category	WHQOL score		Multivariable result	
		Poor QOL	Good QOL	AOR (95% CI)	P-value
Marital status	Married	113 (48.9%)	118 (51.1%)	1	
	Single	57 (54.3%)	48 (45.7%)	1.480 (0.800, 2.739)	0.211
	Divorced	11 (31.4%)	24 (68.6%)	2.921 (1.256, 6.794)	0.013*
	Widowed	8 (29.6%)	19 (70.4%)	2.783 (0.975, 7.948)	0.056
Educational status	Unable to write and read	22 (27.2%)	59 (72.8%)	6.176 (2.872, 13.278)	0.000*
	Primary	45 (39.5%)	69 (60.5%)	3.129 (1.659, 5.900)	0.000*
	Secondary	60 (57.7%)	44 (42.3%)	1.140 (0.593, 2.191)	0.695
	College and above	62 (62.6%)	37 (37.4%)	1	
Relationship with the patient	Parent	35 (43.2%)	46 (56.8%)	1.189 (0.518, 2.730)	0.684
	Spouse	35 (39.3%)	54 (60.7%)	2.407 (1.088, 5.322)	0.030*
	Siblings	52 (51.5%)	49 (48.5%)	2.422 (1.143, 5.135)	0.021*
	Children	31 (50.8%)	30 (49.2%)	2.543 (1.105, 5.853)	0.028*
	Others^d^	36 (54.5%)	30 (45.5%)	1	
Social support	Poor support	76 (35.5%)	138 (64.5%)	3.206 (1.578, 6.511)	0.001*
	Moderate support	68 (56.7%)	52 (43.3%)	1.207 (0.590, 2.468)	0.607
	Strong support	45 (70.3%)	19 (29.7%)	1	
Perceived stigma	Low perceived stigma	134 (57.5%)	99 (42.5%)	1	
	High perceived stigma	55 (33.3%)	110 (66.7%)	2.376 (1.489, 3.792)	0.000*

*p-value < 0.05.

## Discussion

As per the knowledge of the researchers of this study, there is a shortage of literature regarding the quality of life among caregivers of patients with mental illness in developed countries in general and developing countries in Ethiopia in particular. This shortage of evidence makes the treatment of mental illness difficult, creating a huge gap in medication adherence for patients who receive treatment. Therefore, this study assessed quality of life and its associated factors in family caregivers at DRH, Ethiopia.

In this study, the magnitude of poor quality of life was found to be high (47.5%) (95% CI 41.2, 51.5). A study in Uganda showed the quality of life of caregivers and found that 57.3% of the participants had poor quality of life^8^, which is also higher than the current study. Another study conducted in Indonesia found that 53.2% of caregivers had poor quality of life^21^, which is also higher than our finding. Furthermore, studies conducted in Indonesia and Iran found that 36.2%^9^ and 17%^12^ of participants had poor quality of life, which is relatively lower than our study finding. The justification for this difference might be due to the variations in sociodemographic factors, as well as cultural and economic variations between the countries where the current and previous studies were conducted. In addition, methodological variations such as the use of different assessment tools for quality of life and difference in sample size in current and previous studies. Besides, the level of adequacy of mental health care, the different expectations and provisions of mental health care services in these countries, could contribute to the discrepancy.

In this study, there was a significant association between the marital status of the caregivers of patients with mental illness and their QOL. Participants who were divorced were nearly 3 times more likely to have poor QOL than those who were married. This finding is supported by a study conducted in Malaysia, in which being divorced in marital status was significantly associated with poor QOL^24^. Moreover, this study was supported by studies conducted in Iran and Singapore^22,25^. The first possible reason might be that being divorced leads to loneliness and loss of intimate partner relationships, which are major components of QOL. Divorced caregivers might face more challenges in the absence of a spouse who could offer support and share some of the distress.

Moreover, participants who were unable to write and read were 6 times more likely to have poor QOL than those who were educated to college level and above and participants who were educated to primary school level were 3 times more likely to have poor QOL. This finding is supported by a studies conducted in Ghana, Malaysia, Singapore, Hong Kong and India in which highly educated caregivers reported better QOL and having a lower level of education was significantly associated with poor QOL^23–27^. This might be due to the fact that highly educated caregivers have better knowledge of dealing with stressful situations in their life events and good coping mechanisms, which results in better QOL. Highly educated caregivers also tend to have better jobs with higher incomes, which could command more resources that could be helpful in taking care of their ill relatives, which may also contribute to their better QOL. On the other hand, lower education level among caregivers is often related to lower socioeconomic status, which may result in poor QOL.

In addition, participants who were spouses and siblings of patients with mental illness were 2.4 times more likely to have poor QOL, and participants who were children of patients with mental illness were 2.5 times more likely to have poor QOL than other relatives of the patients. This finding is supported by studies conducted in Iran and Singapore that showed that participants who were spouses, siblings, and children were more likely to have poor QOL^22,25^. This might be because many patients with mental illnesses gain support and care primarily from their spouses, children, and siblings. Therefore, due to this fact, these caregivers might have a greater caregiving burden and poor QOL.

Besides, having a poor level of social support was 3.2 times at an increased level to have poor QOL than participants who have strong social support. This finding is inline with other studies conducted in Iran, Indonesia, China and India, in which social support positively affected QOL among family caregivers of patients with mental illness^7,27–29^. It is known that support from others is a fundamental factor for good QOL. However, when this support is compromised, all care responsibilities lie only on the caregivers. This also results in poor QOL among caregivers.

Regarding perceived stigma, having high-perceived stigma was nearly 2.4 times at an increased level to have poor QOL than participants who have low perceived stigma. This finding is supported by the results of studies conducted in Singapore and Egypt showing that participants who have high perceived stigma are more likely to have poor QOL^30,31^. The possible explanation could be that the inability to cope with stigma may result in poor QOL.

## Conclusions

Based on the findings of this study, many family caregivers of patients with mental illness have poor QOL and need special attention. Among the factors associated with poor QOL, marital status (being divorced), educational status (being unable to read and write and educated up to the primary school level), relationship with the patient (spouses, siblings and children), social support (poor support) and high perceived stigma were significantly associated with poor QOL. Therefore, regular evaluation and screening of caregivers' QOL are important for identifying those in need of support. Health care professionals should also be aware of poor QOL caregivers to routinely measure and intervene as needed.

## Data Availability

All data regarding the findings are accessible on reasonable request from the corresponding author.

## Abbreviations

AOR: Adjusted odd ratio
ASSIST: Alcohol, smoking and substance involvement screening test
CI: Confidence interval
CG: Care giver
COD: Crude odd ratio
DCFS: Devaluation of consumer family scale
DRH: Dessie referral hospital
ICCMH: Integrated clinical and community mental health
LAMICs: Low and middle income countries
MSc: Master of science
QOL: Quality of life
PWMI: Patient with mental illness
SPSS: Statistical package for social science studies
WHO: World Health Organization
